# Supplementary material for: Are carbon emissions trading and green financial instruments synergistic? -Comprehensive quantitative research based on content analysis
Source: PLoS One. 2024 Mar 7;19(3):e0298601. doi: 10.1371/journal.pone.0298601 (PMC10919601; doi:10.1371/journal.pone.0298601)
Supplement: S1 Data — (ZIP) [file pone.0298601.s001.zip › Data/地级市Z_F工作报告.zip!/╜¡╬≈/╝¬░▓╩╨/└┤╫╘╣1⁄2╓┌║┼í╛┬φ┐╦╩2╛▌═°í┐_2018.docx]

各位代表：

　　现在，我代表市人民政府向大会报告工作，请予审议，并请各位市政协委员和列席会议的同志提出意见。

　　一、2017年工作回顾

　　刚刚过去的2017年，是党的十九大胜利召开之年，是决战决胜全面小康的重要一年。一年来，在省委、省政府和市委的坚强领导下，全市上下坚持以习近平新时代中国特色社会主义思想为指导，大力践行省委“创新引领、绿色崛起、担当实干、兴赣富民”工作方针，全面贯彻市第四次党代会精神，感恩奋进，砥砺前行，统筹做好稳增长、促改革、调结构、优生态、惠民生、防风险各项工作，较好完成了年度目标任务，迈出了“三个走在前列”“两大战略任务”新步伐。

　　———经济运行稳中向好。经济增速继续保持在全省“第一方阵”，10项主要经济指标增幅位居全省前三。实现生产总值1633亿元、增长9%，高出全省平均水平0.1个百分点。财政总收入251.3亿元、增长9.7%，税占比提高0.3个百分点、达到79%。

　　———经济结构继续优化。三次产业结构为14.9:44.5:40.6，规模以上工业增加值增长9.2%，服务业增加值占GDP比重提高1.3个百分点。社会消费品零售总额505亿元、增长12.6%，消费对经济增长的贡献率提高到52.5%。外贸出口增长14.9%，总量达320亿元、居全省第二。预计高新技术产业增加值增长15%，占规上工业比重提升到40%，高出全省平均水平9.1个百分点。单位GDP能耗全省最低、下降5%。

　　———新动能加快形成。实际新增高新技术企业74家，总数达207家。预计R＆D经费支出①超过13亿元，占GDP比重达0.78%、提高0.2个百分点。新增国家高新技术产业化基地2个、院士工作站7家，新增省级工程技术研究中心4家、省级工业设计中心1家。新登记市场主体增长23.3%，个升企297家、企入规224家。

　　———人民生活持续改善。新增城镇就业5.8万人，新增转移农村劳动力8.2万人，均超额完成年度任务，城镇登记失业率控制在3%以内。居民消费价格总水平上涨1.7%，控制在3%的调控目标内。城镇居民人均可支配收入突破3万元大关，达到31936元、增长9%，农村居民人均可支配收入达到12543元、增长10.2%。

　　回顾过去一年，尤其让我们倍感振奋的是，吉安大地发生了三件大事、喜事。去年2月26日、11月1日，井冈山市、吉安县率先在全国全省实现脱贫摘帽；9月19日，吉安市获评全国社会治安综合治理优秀市；11月14日，我们首次创建、一举获得全国文明城市最高荣誉，创出了自信心、自豪感，锤炼出了“有志者事竟成”的创城意志力。成绩来之不易，这是在国内外经济趋紧的宏观环境下取得的，是在全省各地竞相发展的外部条件下取得的，也是在我市诸多矛盾不断凸显的情况下取得的。在困难和压力面前，全市人民弘扬井冈山精神，迎难而上，攻克一个又一个难关，取得一个又一个胜利。这也再次表明，任何困难，都抵挡不住530万吉安人民拥抱新时代、创造新生活的坚定决心和坚实步伐！

　　一年来，我们主要做了以下工作：

　　一是综合施策稳增长，经济实现较快发展。坚持把稳增长作为首要任务，多措并举推动经济中高速增长。聚焦项目建设，成功争取52个重大项目和平台获得国家部委支持，56个项目纳入全省新一轮苏区振兴发展重大事项；广吉高速等400个重点项目扎实推进，井冈山航电枢纽工程开工建设，“点亮吉安”等7个PPP②示范项目进展顺利，全年完成固定资产投资1930亿元、增长12.9%，其中民间投资增长18%。聚焦实体帮扶，深化“降成本、优环境”专项行动，“四定四零”③帮扶举措在全省推广，全年为企业减负80.6亿元；把人才培养作为根本举措，与长江商学院合作举办“领航井冈”企业家高级研修班，7位吉安企业家入选全省回乡投资优秀赣商。聚焦全面开放，举办返乡兴业、电子信息、大健康、文化旅游、现代农业等招商活动117场次，股权招商再创佳绩，IPO④拟上市总部企业招商取得突破，吉安至深圳盐田港铁海联运快速班列正式开通，全市实现签约项目、合同引资额、招大引强“三个翻番”，引进内资656.9亿元、增长12%，实际利用外资10.7亿美元、增长9.8%，新签约项目295个，其中10亿元以上重大项目47个。

　　二是坚定不移调结构，质量效益稳步提升。坚持把优结构、转方式作为主攻方向，久久为功促进转型升级。推动工业量质齐升，首位产业和龙头企业“优惠八条”⑤全面落实，电子信息产业研究院正式成立，首个百亿项目益丰泰TFTLCD显示面板项目在吉安落地，合力泰成为全市第一家过百亿企业，航盛电子获省长质量奖提名奖，电子信息产业主营业务收入突破800亿元；大健康产业全面布局，康养旅游、中医药等细分产业竞相发展。推动现代农业提质增效，粮食生产实现“十四连丰”，总产达84亿斤，收购量全省第一；新增六大富民产业⑥70.3万亩，井冈蜜柚获国家地理标志证明商标，首次出口俄罗斯、香港、澳门等国家和地区；40.5万亩高标准农田开工建设，全市新型农业经营主体达1.4万家。推动现代服务业提速升级，“全景吉安、全域旅游”战略深入实施，“红色摇篮、山水吉安”品牌宣传在央视重磅推出，荣获全国旅游“厕所革命”先进市，发放旅游惠民卡6万张，井冈山机场旅客吞吐量突破60万人次，安福羊狮慕、万安田北农民画村成功创建国家4A级景区，井冈山旅游公司获“中国质量奖提名奖”，井冈山神山村、遂川桃源梯田、泰和蜀口生态岛等成为旅游新亮点，吉安旅游迎来了“百花齐放”新格局，全市接待游客7974.7万人次，实现旅游总收入763.2亿元，分别增长28.5%、38.6%；创立全国第一家红色金融博物馆，全市金融机构新增贷款287亿元，增幅居全省第二，存贷比提高4个百分点；文化创意、共享经济等新业态新模式蓬勃兴起，实现电商产业园县域全覆盖、电商服务站乡镇全覆盖。推动区域协调发展，井冈山经开区工业主营业务收入突破600亿元、挺进全省十强，吉安高新区在全国排名上升十二位，吉泰走廊生产总值占全市比重提高到53.5%；各县市区的财税质量进一步提升，新增泰和触控显示百亿集群，新干箱包纳入国家区域品牌建设试点。

　　三是深处着力抓改革，动力活力集聚释放。推进供给侧结构性改革，打好“三去一降一补”攻坚战，关闭退出煤矿企业3家，取缔“地条钢”4家，全面完成去产能年度任务。推进政府“放管服”改革，精简行政审批事项36项、其他权力事项102项，取消和调整行政审批事项82项；“24证合一”⑦“一照一码”登记制度改革全面完成，“双随机一公开”监管实现全覆盖；吉州区“一个电话服务到、一枚公章审批完”改革成效明显；智慧城市建设加快推进，数据采集基本到位，手机APP上线运行。农地确权全面完成，农村集体产权制度改革扎实推进。公立医院综合改革、县域城乡义务教育一体化改革、中心城区城市执法体制改革全面推开。

　　四是统筹城乡促融合，环境面貌有效改善。加大城市建设力度，组建中心城区五大城建重点项目指挥部，市领导领衔担当，各方合力共为，高铁新区建设实现土地征用、房屋征收全启动，第一批9个项目当年谋划当年开工，完成投资10亿元；吉安大桥西岸互通工程提前开工、破难而进；畅通“微循环”项目进展迅速，一年打通“断头路”48条；赣西堤开发纵深推进，神岗山大桥开工建设；棚户区改造新开工4520套；完成美丽乡村点建设4431个，全市25户以上自然村基本实现通水泥路，吉安县永和镇被列为全国特色小镇，全市城镇化率提高1.2个百分点、达到49%。高效能加强城市管理，“数字城管”二期建成投用，网格化管理全面推行，“重心下移、综合执法”城管格局加快形成。重拳整治城乡环境，超店经营、乱搭乱建等专项整治行动深入开展，累计拆除“两违”建筑150多万平方米；农村生活垃圾专项治理成效明显，所有县市区通过省级考核验收；樟山垃圾处理场渗滤液处理站改造基本完成，中心城区生活垃圾焚烧发电项目稳步实施，吉安顺利通过国家卫生城市复审。

　　五是聚焦聚力优生态，绿色优势巩固提升。积极融入国家生态文明试验区建设，六大制度体系、六大重点工程扎实推进。耕地保护有效加强，生态保护红线基本划定，占国土总面积的23.9%，实现生态管控“一张图”。“三禁”工作⑧开局良好，清土封尘、秸秆禁烧，以及“四尘”“三烟”“三气”⑨等专项整治力度之大前所未有，中心城区燃煤锅炉全面关停退出，空气质量保持优良。“河长制”实现全覆盖，劣Ⅴ类水全面消灭，主要河流监测断面水质达标率100%。生猪生态循环养殖小区建设加快推进，“禁养区”全面退养。完成人工造林29.5万亩、森林抚育32.1万亩，遂川南风面获评国家级自然保护区，罗霄山大峡谷获批国家森林公园，全市新增2个省级生态文明示范县，新增3个工业园区纳入省级循环化改造试点。领导干部自然资源资产离任审计试点扎实推进。全市新投放新能源公交车222辆、公共自行车6000多辆，有力促进了低碳出行、健康生活。

　　六是不遗余力惠民生，百姓福祉持续增进。全市民生投入353亿元，占财政总支出比重提高到81.2%。省市95件民生实事全面完成。脱贫攻坚取得重大阶段性胜利，减贫5.2万人、195个贫困村退出，全市贫困发生率降至1.2%，老区贫困群众激发“我要脱贫”的斗志，摆脱困境、共奔小康。筹资38亿元，将12.95万被征地农民纳入基本养老保障，解决了近二十年的历史遗留问题。成功争取全国居家和社区养老服务改革试点。企业退休人员养老金“十三连增”。教育事业稳步发展，新增学前教育学位1.8万个，“一乡（镇）一所”公办幼儿园建设基本实现，新增吉水、万安和井冈山市通过国家义务教育均衡发展督导评估，高考再创佳绩。卫生事业加快发展，城乡居民基本医疗保险、大病保险实现全覆盖，贫困患者医疗费用自负率控制在8%以内。文化惠民工程扎实推进，成功举行井冈山革命根据地创建90周年纪念活动，吉州窑入选国家考古遗址公园。吉安市第五届运动会顺利举行，第18届亚洲跳伞锦标赛暨中国跳伞公开赛落户吉安，井冈山红色国际马拉松赛成功举办。平安吉安、法治吉安建设深入推进，食药安全、信访维稳、安全生产形势总体平稳，安全生产“两张表”輥輮訛实现全覆盖，公众安全感继续位居全省前列。

　　一年来，我们大力加强政府自身建设，提升服务水平。推进依法行政，坚持市政府常务会学法制度，出台《吉安市人民政府规章制定程序规定》，推动《吉安市城市市容和环境卫生管理条例》颁布实施，提请审议《吉安市水库水质保护条例》。推进民主决策，严格履行公众参与、专家论证、风险评估、合法性审查、集体讨论行政决策程序。推进廉洁建设，严格落实中央八项规定精神和国务院“约法三章”，加强行政监察、审计监督、投资评审，市本级“三公”经费下降23.3%。

　　各位代表，这些成绩的取得，是习近平总书记亲切关怀和党中央、国务院特殊支持的结果，是省委、省政府和市委坚强领导的结果，是全市人民团结拼搏的结果，是社会各界关心支持的结果。在此，我代表市人民政府，向全市广大干部群众，向各民主党派、工商联、无党派和社会各界人士，向驻吉人民解放军、武警官兵和公安消防干警，向所有关心支持吉安发展的同志们、朋友们，致以衷心的感谢和崇高的敬意！

　　需要说明的是，去年全市规模工业增加值、固定资产投资、农村居民人均可支配收入等指标增长低于预期目标。这既是受宏观经济和调控政策等客观因素的影响，更与我市经济社会发展自身存在的困难和问题直接关联。我们清醒认识到，我市经济总量不大、人均GDP偏低，产业层次不高、创新能力不强、大项目不多，品牌响、质量优、市场好的产品比较缺乏，实体经济仍然面临不少困难；环境保护与新时代要求还有较大的差距，住房、教育、医疗、养老等方面还有不少短板；群众办事难问题仍较突出，政府少数干部思想保守、理念落后、缺乏担当、落实不力，政府效能有待进一步提高。对此，我们一定直面问题，敢于担当，全力以赴做好各项工作，不辱历史使命，不负人民重托！

　　二、2018年工作安排

　　今年是贯彻党的十九大精神的开局之年，是改革开放40周年，是决胜全面建成小康社会的关键一年，也是吉安加快绿色崛起、开启现代化征程打基础的重要一年。做好政府各项工作十分重要。今年政府工作总体要求是：深入贯彻落实党的十九大、中央经济工作会议、省委十四届五次全会和市委四届五次全会精神，以习近平新时代中国特色社会主义思想为指导，大力弘扬跨越时空的井冈山精神，按照高质量发展的要求，坚持新发展理念，坚持稳中求进工作总基调，坚持以供给侧结构性改革为主线，着力构建现代化经济体系，着力深化改革开放，着力加强生态建设与环境保护，着力打好脱贫攻坚战，着力保障和改善民生，促进经济社会持续健康发展，践行“三个走在前列”，实施“两大战略任务”，奋力迈出谱写新时代中国特色社会主义吉安篇章的坚实步伐！

　　今年全市经济社会发展的主要预期目标是：生产总值增长9%左右，财政总收入增长9%左右，规模以上工业增加值增长9%左右；固定资产投资增长11.5%，社会消费品零售总额增长12.5%，实际利用外资增长9%左右，外贸出口总额增长3%左右；城镇和农村居民人均可支配收入分别增长8.6%和9.5%左右，居民消费价格上涨3%以内，城镇登记失业率控制在4.5%以内，节能减排完成省下达任务。

　　工作中，我们将做到“四个牢牢把握”：一是牢牢把握新时代社会主要矛盾的变化，紧扣社会需求的特点，在供给侧结构性改革上下功夫，全面提高产品和服务的供给质量，努力提升人民群众的获得感、幸福感、安全感。二是牢牢把握当前发展阶段由高速增长转向高质量发展的特征，在推进质量第一、效益优先、结构优化的过程中，稳增长、调结构、提质量，推动质量变革、效率变革、动力变革。三是牢牢把握全面建成小康社会的总体目标，系统对标，查补短板，既突出抓好脱贫攻坚，又实现各项事业整体推进，注重系统性、协调性，为决胜全面小康社会迈出坚实步伐。四是牢牢把握治理体系和治理能力现代化的任务要求，把完善社会治理结构的要求落实到工作中，在夯实基层基础上下功夫、创特色、见成效。

　　围绕上述目标，重点抓好以下九个方面的工作：

　　（一）深入推进供给侧结构性改革，加快推动产业迈向中高端。坚持向特色优势要竞争力，加快构建富有吉安特色的现代化产业体系，不断提高供给质量和效益。

　　强化科技创新。发挥政府引导作用，落实专项专利补助、企业研发费用税前加计扣除优惠政策，鼓励企业设立研发准备金，力争全社会R&D经费支出占GDP比重达到1%。发挥企业主体作用，实施10个市级科技协同创新重大专项、8个重大科技研发专项，推动泰和、安福和永丰工业园区创建省级高新技术产业园区，新增一批省级以上企业工程技术中心。发挥平台支撑作用，全面运作电子信息产业研究院、院士工作站，加快生产力促进中心建设，承办第二届国际存储与计算研讨会，加强校企对接，推进协同创新，促进科研成果在吉安转化应用。

　　聚焦“1+4”产业。集聚优势资源，提供优质服务，坚定不移打造“1+4”工业輥輯訛“头雁方阵”。做强电子信息首位产业，设立运作百亿规模引导基金，争取纳入省级基金群；实施LED智能照明和移动通讯终端两大集群链接工程，推进益丰泰TFTLCD面板、欧司朗照明、立讯射频等项目建设，推动合力泰总部迁入吉安，促进电子信息产业“点、线、面、体”全链发展，跨入千亿产业行列。加快发展先进装备制造业，实施“制造业50强培育计划”，争创“中国制造2025”国家示范区。做大生物医药大健康产业，规划建设一批县域生物医药产业园，推动国定贫困县纳入国家中医药发展“三定三百”工程輥輰訛实施范围，全市新增特色药材种植面积3万亩；布局建设吉州樟山新区、井冈山市柏露等一批温泉养生、康养休闲基地。

　　培育新兴产业。深入实施“全景吉安、全域旅游”战略，推动吉安旅游投资公司全面运作，打造集“景区建设、品牌提升、市场培育、产业发展”为一体的旅游集团，带动旅游资源系统整合、集成开发；加快15个市级旅游集聚区建设，启动吉州窑申报创建国家5A级景区，提升A级景区品质，建设蜀水河生态旅游区，构建环中心城区旅游圈；发展乡村旅游，开发精品民宿，促进农旅结合、文旅融合，形成“三区一网”輥輱訛旅游发展格局，将旅游产业打造成为全市支柱产业。积极布局通航产业，实施桐坪机场改扩建和快速通道建设，推动桐坪机场纳入国家航空运动试点，打造桐坪航空小镇，加快安福、遂川通用机场建设，推进武功山国家通航旅游示范工程。发展军民融合产业，发挥国防科工局全行业对口支援优势，培育军民融合企业，加快井冈山经开区和吉水县省级军民融合产业基地建设，争创国家军民融合创新示范区，推动军民融合向全要素、多领域、高效益发展。

　　提升传统产业。坚决淘汰落后和过剩产能，关闭退出8家煤矿，完成43万吨煤矿去产能任务，杜绝新增过剩产能，严防“地条钢”死灰复燃。实施企业技改深化年行动，增强企业新产品、新技术、新工艺开发应用能力，推动食品、建材等传统产业向智能化、绿色化方向发展，实现“老树发新芽”。

　　（二）强化实体经济和有效需求支撑，不断筑牢持续健康发展的坚实根基。坚持供需双侧发力，做强实体、扩大需求，推动经济行稳致远、提质增效。

　　壮大实体经济。抓帮扶、强实体，全面落实国家新一轮减税降费政策和省市“降成本、优环境”扶持措施，进一步清理涉企政府性基金和事业性收费，规范“五险一金”缴费，组织扩大直购电用户参与范围，不断降低企业用工和要素成本。抓金融、活实体，增加金融机构，丰富金融业态，推进融资担保平台县域全覆盖，做大工业投融资平台，注册设立全国性农业专业保险公司，争创国家普惠金融改革试验区，推进企业上市“115”工程輥輲訛，力争全市金融机构新增贷款300亿元、企业直接融资100亿元以上。抓服务、促实体，加快发展工业设计、第三方物流、融资租赁、检验检测等生产性服务业，完善实体经济服务链。

　　扩大有效投资。重点围绕基础设施、产业升级、民生改善、生态环保和城市建设等领域，安排360个重点项目，年内完成投资900亿元以上。加快昌吉赣客专、蒙华铁路、亚行贷款城市综合交通等在建项目建设，实施赣江大桥危桥改建，完成永丰高龙山、万安高山嶂等一批清洁能源项目建设。加大融资力度，争取政策性金融机构专项基金和债券投资，继续推出一批PPP示范项目，激发民间投资活力。强化争项争资，跟踪对接国家新一轮苏区振兴政策，谋划一批重大项目，力争更多项目进笼子、更多资金进盘子。

　　挖掘消费潜力。深化“增品种、提品质、创品牌”专项行动，加强特色品牌包装和宣传，优供给、促消费。增加无线网络、数字电影、动漫游戏等信息产品供给，促进信息消费。落实新能源汽车发展扶持措施，加快汽车充电桩和快充网建设，促进节能环保汽车消费。实施“互联网+流通”行动计划，发展自助商业模式，推进大型商场、高端超市等商业综合体建设，打通“农货进城”“网货下乡”双向通道，全面带动城乡消费。加强质量监管，强化商标、品牌引领，健全消费者权益保护机制，让群众放心消费、安心消费。

　　推动外贸升级。优化外贸结构，引进和扶持生产型出口企业，扩大机电、高新技术和农特产品出口，发展服务贸易。完善井冈山出口加工区功能，创建国家电子信息产业出口质量安全示范区，复制国家自贸区经验，推广国际贸易“单一窗口”輥輳訛模式，争创国家跨境电商综合试验区，发展外贸新业态。

　　（三）坚定实施重点板块带动，促进区域城乡协调发展。抢抓高铁即将开通、苏区振兴重要“窗口期”，抓重点、带全面，加快形成区域协同、城乡融合新格局。

　　推动吉泰走廊升级发展。用好用活省级协调机制，全面落实省政府支持走廊建设会议精神，强化吉泰走廊的脊梁地位。创新体制机制，推进井冈山经开区体制改革，实现与吉州工业园、河东工业园、富滩工业园融合发展，完善产业定位，突出功能分区，打造全省战略性新兴产业集聚区，迈向千亿园区；推进吉安高新区完善配套、集聚项目、创新发展，争创国家文化科技融合示范基地和循环经济示范园区，完成主营业务收入420亿元；推动吉泰走廊产业差异化布局、联动式发展，打造吉泰走廊先进制造业样板区、现代农业示范区和现代服务业集聚区。加快走廊基础设施升级，推进105国道吉水至泰和段改造工程，争取吉安至井冈山客专项目立项，启动吉泰城镇群快速通道建设，促进城镇组团融合互动。加快走廊服务升级，完成华能电厂园区供热工程，实施天然气管网延伸项目，促进公共服务一体化。

　　推动高铁经济带加快发展。加强高铁经济带规划研究，举办高铁经济高峰论坛。推进中心城区高铁新区“龙头工程”综合开发，基本完成站前广场、站前大道建设，谋划实施人才科创中心、电子信息研发基地、高端住宅和城市综合体等重大项目，推动高铁新区主体框架基本成型。纵深推进昌吉赣、渝长厦高铁沿线谋篇布局，全面加强沿线村庄集镇整治，主动谋划各地高铁新城建设，管控土地开发，统筹产业布局，努力打造吉安高铁主轴经济带。

　　推动县域经济均衡发展。加强县域首位产业的选择、论证，引导各县市区科学确立首位产业、主导产业，形成“1+N”产业定位。完善县域发展支持体系，在融资、扩权、用地等方面进行差异化倾斜，支持新干盐卤药化、峡江生物医药、永丰循环经济、遂川硅基材料等特色产业做大做强，推动永丰铁路产业园建设，打造吉州区通讯传输百亿产业集群。抓好省直管县试点，探索经济发达镇管理新体制，推动县域经济向城市经济、都市区经济转型。

　　推动新型城镇化提档升级。着力优化布局，加快中心城区城南、赣西堤和滨江新区综合开发，提升庐陵新区核心带动力；推动县城提质扩容，打造吉州窑陶艺小镇、罗浮双养小镇、羊狮慕康养小镇等特色小镇，形成三级梯度城镇格局。着力完善功能，加快吉安大桥西岸互通、神岗山大桥、禾河大道路堤结合改造工程建设，启动中心城区滨江大道北延工程，中心城区年内再打通27条“断头路”，新建17个停车场和一批标准化菜市场；推进“城市双修”輥輴訛和“海绵城市”建设，加快河东滨江生态公园建设，新建一批街头小游园和公共绿地。着力创新管理，宣传执行《吉安市城市市容和环境卫生管理条例》，推动颁布《吉安市物业管理条例》和《吉安市绿地管理条例》，提高城市管理法治化和精细化水平。着力提升品质，实施背街小巷和老旧小区“三改三通三进”三年行动计划，路面改沥青、屋顶改违搭、生活改陋习，疏通下水道、连通绿化带、打通断头路，推动配套设施进小区、公共服务进小区、文明新风进小区。着力推进文明城市长效常态、巩固提升，支持安福县、吉水县创建国家卫生城市，支持新干县、井冈山市创建全国文明城市，深化文明村镇、文明单位、文明校园、文明家庭创建，常态化开展文明市民评选活动，让文明市民成千上万，让文明行为蔚然成风。

　　（四）纵深推进重点改革，不断释放发展动力和活力。聚焦重点领域，强化改革攻坚，突破体制桎梏，进一步激发市场活力和社会创造力。

　　深化“放管服”改革。着力增强放权的协同性，进一步取消或下放一批行政权力，公布乡镇政府权责清单，实现市县乡三级政府权责清单全覆盖；推进相对集中行政许可权改革，全面实行“多证合一、一照一码”制度，抓好“证照分离”试点。着力增强监管的针对性，健全协同监管和联合惩戒机制，完善诚信档案、黑名单制度；加强市县执法机构整合，探索乡镇街道“一支队伍管执法”，实现相对集中执法“多帽合一”輥輵訛。着力增强服务的可及性和便捷性，推进“一次不跑”改革，开展“减证便民”行动，继续抓好国家智慧城市试点，全面对接江西政务服务网，努力实现群众和企业办事“单点登录、全程网办”。

　　推进开发区改革创新。打破行政体制壁垒，强化经济管理、招商引资和投资服务功能，提升工作效能，实现“园区事、园区办结”。创新营运机制，剥离社会事务，推行“以岗定薪、按绩计酬”的薪酬绩效制度，支持开发区设立具备独立法人资格的投融资平台。优化用地布局，严格执行工业用地投资强度标准，加强地上地下综合利用，加大批而未用、闲置土地和“僵尸”企业清理力度，新建标准厂房100万平方米，推动开发区集约发展、提档升级。

　　统筹推进重点改革。推进盐业体制改革，完成市国资、国投公司整合，实现国有资产监管全覆盖。深化财税体制改革，实施绩效管理，规范政府举债，消化存量债务，严控增量债务，防范债务风险；促进省市县治税平台互联互通。加快事业单位分类改革，推动符合条件的事业单位转为企业或社会组织，激发事业单位活力。

　　（五）持续扩大全面开放，大力拓展发展新空间。放宽视野，放活思维，积极融入全球开放发展大格局，迈出双向开放、全面开放新步伐。

　　拓展开放区域。融入“一带一路”，加强同沿线国家和地区的经贸合作，开展“百企出境参展”活动，力争实现出口企业“零费用”参展，推动“吉安制造”“吉安产品”走出去。参与长江经济带建设，加快新干、井冈山航电枢纽工程建设，启动泰和沿溪、吉水醪桥、峡江货运码头等港口建设，推进赣江石虎塘至神岗山Ⅲ级航道整治工程，提高赣江吉安段通航能力。对接京津冀，融入“长珠闽”，密切吉港经贸活动，推进赣台深化合作。

　　拓宽开放领域。坚持招商引资“第一菜单”，实施招大引强、返乡兴业、利用外资、首位产业等系列攻坚行动。巩固工业招商，拓展农业招商，加强旅游康养、文化教育等服务业招商，促进三次产业全面开放。紧盯世界500强、港台200大、民企500强，引进一批补链、强链、延链型项目和总部企业。

　　提升开放优势。进一步畅通对外通道，启动大广、樟吉高速吉安段“四改八”工程，完成宜井遂高速宜春至井冈山段前期工作；做好吉抚武温、新余至吉安城际铁路前期工作，推动长赣高铁年内开工；完成井冈山机场二期扩建工程，新开通昆明至上海浦东等经停航线。持续优化软环境，强化“店小二”“保姆式”服务，构建“亲”“清”新型政商关系，让每个在吉安投资的客商既能看到笑脸，又能看到效率。

　　（六）大力实施乡村振兴战略，加快推进农业农村现代化。坚持农业农村优先发展，按照产业兴旺、生态宜居、乡风文明、治理有效、生活富裕的总要求，集成资源、集聚力量，推动农业全面升级、农村全面进步、农民全面发展。

　　提升现代农业发展水平。坚持质量兴农、绿色兴农，确保粮食安全，壮大特色产业，新增六大富民产业种植面积40万亩以上，形成“产地生态、产品绿色、产业融合、产出高效”的现代农业发展新格局。推进规模化布局，落实第二轮土地承包到期后再延长30年的政策，完善承包地“三权分置”制度，加快农村产权交易市场建设，年内再完成27万亩高标准农田建设，推行政府（集体）“兜底收储、规模流转”方式，促进土地向龙头企业、专业合作社、家庭农场等新型主体有序流转；推动井冈山国家农业科技园创建国家级农业高新技术产业示范区，新增一批省级现代农业示范园，建设一批三产融合田园综合体。推进标准化生产，实施柑橘类黄龙病防治计划，率先实现吉泰粮食主产区机械化作业。推进生态化发展，持续开展绿色生态农业“十大行动”，全面淘汰小规模生猪养殖，建成30个以上生态循环养殖小区，打造“稻虾共生”等综合种养基地2万亩以上。推进品牌化经营，强化“井冈山”绿色农产品公用品牌建设，提升井冈蜜柚节、狗牯脑茶文化节影响力，延伸泰和乌鸡、安福火腿等传统特色产业新链条。健全信息、农技、气象等综合服务体系，推进“123+N”智慧农业輥輶訛建设，加强农田水利设施建设，基本完成南车灌区续建配套与节水改造。

　　加快推进农村现代化。深化“整洁美丽、和谐宜居”新农村建设，高标准完成3800个美丽乡村点建设，美丽乡村覆盖率达90%以上。推动城乡融合发展，促进公共服务和基础设施向农村延伸，全面解决农村饮水安全问题，加大农村“改厕”力度，完成农村公路改造700公里、危桥改造140座，新增一批“四好农村路”輥輷訛。发展村级集体经济，基本消除年经营性收入低于2万元的“空壳村”。实施乡村记忆工程，加强传统村落保护，建设一批“乡村博物馆”“村情展示馆”。推进移风易俗，健全乡规民约，完善自治、法治、德治相结合的乡村治理体系。

　　大力培育新型农民。加强职业农民和农业职业经理人培训，推动农民工、中高等院校毕业生、退伍军人、科技人员加入职业农民队伍，鼓励支持农村外出务工人员返乡创业做大“归雁经济”。做好村（居）委员会换届工作，吸引更多新型农民进入村级班子，造就一支懂农业、爱农村、爱农民的“三农”工作队伍，以农村的人气指数引领农村的发展指数。

　　（七）切实加强生态文明建设，稳步提升生态环境质量。大力践行绿水青山就是金山银山理念，推进国家生态文明试验区建设，聚焦制度创新，巩固生态优势，加快迈向生态文明新时代。

　　打好环境污染防治攻坚战。坚决抓好中央和省环保督察反馈问题整改，对标更严的标准，采取更实的举措，推动环保工作全面加压，促进生态环境质量稳中有升。开展第二次全国污染源普查，健全环境信息平台，全面监测与调度大气、水、土壤等环境质量指标。深入开展“守护蓝天”行动，继续抓好工业废气、汽车尾气、油烟直排、工地扬尘等污染防治，全面推进“三禁”工作，加强农作物秸秆禁烧和综合利用，建立空气质量预警响应和区域环评机制，精准严格，科学施策，打赢蓝天保卫战。深入开展“清河护河”行动，打造河长制“升级版”，全面消灭城区黑臭水体，对一切非法采砂和库区违占等破坏河道岸线的违法行为严厉打击、决不手软；启动中心城区备用水源项目建设，保障群众用水安全。加强工业园区污染防治，所有园区建成污水集中处理设施并实现在线联网、实时监测，推动井冈山经开区创建国家生态示范园区。加快土壤污染修复，加强农业面源污染防治，保持农药化肥使用量“零增长”。

　　实施山水林田湖草保护与建养工程。严守生态保护红线，对红线区域严格管控。开展国土绿化行动，提升森林质量，完成人工造林22万亩、低效林改造19.6万亩、森林抚育25万亩，抓好国开行贷款国家储备林基地建设，发展特色林下经济。稳定林地湿地保有量，加强国家湿地公园和风景名胜区管理。推进赣江水生态综合治理与修复工程，打造赣江最美岸线。

　　推动城乡环境整治常态化。完成县市区垃圾焚烧发电项目选址规划，开工建设中心城区垃圾焚烧发电项目；启动螺子山污泥处理厂和生活污水处理厂二期建设，兴建一批建筑垃圾消纳场，完成樟山垃圾处理场改建扩容和危弃物处置中心建设，所有中心乡镇建成标准化垃圾压缩站。深化专项整治，加强农村建房管控，确保农村违规建房“零增长”；推进殡葬改革，继续整治“三沿六区”乱埋乱葬现象，加快吉州区城市公益生态墓园建设。健全长效机制，抓好中心城区、井冈山市垃圾强制分类试点，逐步在全市推开；完善乡镇环境重点管理制度，抓好第一批13个环境整治末位乡镇问题整改；全面推行农村垃圾第三方治理，促进环卫治理城乡一体化，打造“清、净、整洁”“精、细、秀美”的城乡环境。

　　创新完善生态制度体系。建立自然资源确权登记系统，开展自然资源资产负债表编制试点，坚决纠正“资源低价、环境无价”的错误认识。完善环保监管体系，加快组建吉安市国有自然资源资产管理机构，完成环保机构监测监察执法垂直管理，促进环境保护统一监管、综合执法；开展生态护法行动，加快设立生态法庭，对破坏生态环境的违法行为做到严判快结。全面落实自然资源资产离任审计制度，细化生态环境损害责任追究办法，完善生态补偿机制。依托中科院泰和千烟洲试验站，组建吉安生态环境研究院。弘扬生态文化，倡导低碳生活。

　　（八）织密扎牢民生保障网，持续增加人民群众获得感。抓住人民群众最关心最直接最现实的利益问题，尽力而行，量力而为，一件接着一件办，一年接着一年干，让人民获得感、幸福感和安全感更加充实、更有保障、更可持续。

　　坚决打赢脱贫攻坚战。着力扩大战果，聚焦深度贫困地区和深度贫困人口，采取更有力的举措、更集中的支持、更精细的工作，确保全市农村贫困人口再减少2.3万人以上，贫困村退出80个以上，万安县、永新县通过国家脱贫退出第三方评估，遂川县实现脱贫摘帽，全市现行标准下95%以上的农村贫困人口稳定脱贫，夺取脱贫攻坚战决定性胜利。着力提升质量效果，坚持志智双扶，激发贫困人口内生动力；推进产业扶贫全覆盖工程，发展“产业扶贫信贷通”，推广“扶贫车间”，实现光伏扶贫全覆盖；实施健康扶贫再提升工程，筑牢“四道防线”輦輮訛，探索慈善“爱心”救助机制，推行县域内定点医院住院“先诊疗后付费”和医疗费用补偿“一站式”结算服务，让贫困户最大限度、最快速度报销医疗费用；统筹推进教育扶贫、易地扶贫搬迁、保障扶贫和村庄整治再推进工程。着力巩固成果，健全长效机制，坚持队伍不撤、投入不减，稳定井冈山市、吉安县脱贫成效，推动脱贫村、脱贫人口向致富文明的更高目标迈进。同步推进城镇困难群众精准帮扶、脱贫解困工作，突出就业援助和兜底保障，完善志愿服务、慰问走访关怀机制，倡导社会扶贫、行业扶贫，让每个困难群众共享美好生活。

　　促进更充分和更高质量的就业。落实积极就业政策，做好高校毕业生、农民工、下岗职工等重点群体就业工作，深化大学生就业创业计划，抓好新干县、遂川县、泰和县和井冈山市国家级农民工返乡创业试点，全市新增城镇就业4.5万人、转移农村劳动力6万人。坚持以创业带就业，新增创业担保贷款10亿元，力争每个县市区建成1个以上省级创客空间或孵化基地。强化就业培训和创业辅导，加快实训基地建设，落实农民工工资保障金和应急周转金制度，维护劳动者合法权益，让广大群众在劳动中创造财富、在奋斗中实现价值。

　　完善更高水平的社会保障体系。强化住房保障，健全房地产长效稳控机制，推行年度住宅用地出让计划公开公示制度，稳定信心，稳住预期，稳控房价；探索开发长租公寓、共有产权住房，落实“租购同权”輦輯訛，培育住房租赁企业，新开工改造棚户区20280套，加快建立多主体供给、多渠道保障、租购并举的住房制度。大力发展养老事业，抓好全国居家和社区养老服务改革试点，完善城乡居民基本养老保险制度，对接养老保险全国统筹；推进医养结合、学养结合，探索建设养老大学，新建一批农村养老院。提高城乡低保对象和特困人员供养标准，加强基层民政能力建设，完善城乡低保动态管理机制。

　　（九）协调推进社会事业，不断满足人民群众的美好生活需要。始终坚持以人民为中心的发展导向，加快补齐公共服务短板，推动各项社会事业全面进步。

　　坚持教育优先发展。落实立德树人根本任务，发展素质教育，加强创新教育，办好人民满意的教育。推动学前教育普惠发展，全面完成乡镇公办幼儿园建设并规范办学，启动中心城区城南片区公办幼儿园建设。推动义务教育均衡发展，扩大城区办学规模，加强农村薄弱学校改造，逐步解决“城镇挤、乡村弱”“大班额”“择校热”等问题，确保所有县市区通过国家义务教育均衡发展督导评估。实施高中阶段教育普及攻坚计划。支持井冈山大学申报硕士学位授予权单位，优化吉安职业技术学院专业设置，筹建吉安铁道职业技术学院和吉安幼儿师范高等专科学校。支持社会力量办教育，推进私立学校、双语学校建设，满足人民群众个性化、高端化的教育需求。

　　加快健康吉安建设。深化公立医院改革，完善分级诊疗制度，扩大家庭医生签约服务覆盖率。加强残疾人康复中心建设，推进市儿童医院全面运营，实施县级医院、乡镇卫生院标准化建设，实现标准化村卫生室全覆盖。加强全科医生队伍建设，为群众提供全方位全周期的健康服务。开展健康素养促进行动，加强卫生防疫工作。努力创建省级食品安全示范城市。

　　繁荣发展文体事业。深入实施文化强市战略，推进国家庐陵文化生态保护区建设，加强庐陵文化、王阳明与吉安等传统文化研究，强化庐陵文化生态园的文化展示功能，提升白鹭洲书院、阳明书院陈展，推进吉水进士博物馆建设。加强旧志整理，加大非物质文化遗产传承保护。加强红色文化、现实题材文艺创作，筹办全市第六届小戏展演。抓好哲学社科普及工作。开展全民健身活动，备战第十五届省运会，筹办中心城区国际马拉松赛。

　　加强和创新社会治理。以“法安天下、德润人心”理念推进法治吉安建设，弘扬社会主义核心价值观，培育自尊自信、理性平和、积极健康的社会心态。健全重大决策风险评估和涉稳问题分类归口处置机制，完善调解仲裁、复议诉讼纠纷化解机制，认真办理群众来信来访，深化“雪亮工程”輦輰訛，完善治安防控体系，实现预防、化解、管控一体化。实施“人文社区·温馨家园”提升工程，发挥社会组织作用，促进政府治理和社会调节、居民自治良性互动。弘扬生命至上、安全第一的思想，严格落实安全生产责任制，坚决防止重特大事故发生。加强应急体系建设，提高防灾减灾救灾能力，确保社会长治久安、人民安居乐业。

　　各位代表！新时代新使命，更加需要军政军民大团结。我们要大力支持国防和军队改革建设，做好国防动员、国防教育、人民防空和优抚安置等工作，开展新一轮全国全省“双拥”模范城创建。大力支持工、青、妇、红十字会等群团组织建设，加强民族宗教工作，扎实做好外事、侨务等各项工作。

　　各位代表！功以才成，业由才广。人才是最大的发展变量，也是最具竞争力的战略资源。我们将大力实施人才兴市战略，打造转型升级“强引擎”。加大引才力度，构建各类功能性创新研发平台，推进人才公寓建设，完善人才服务机制；发挥吉安赣商联合会纽带作用，吸引更多吉安赣商赣才返乡兴业。加大育才力度，实施企业家成长计划，继续办好“领航井冈”企业家高级研修班，设立企业贡献奖，壮大技能人才队伍，弘扬企业家精神、工匠精神和劳模精神，让各类人才在庐陵大地活力迸发、才智涌流。

　　各位代表！面向新时代，踏上新征程，政府工作要有新气象新作为。我们将牢固树立“四个意识”，坚决维护习近平总书记党的领袖和核心地位，扎实开展“不忘初心、牢记使命”主题教育，努力做到信念过硬、政治过硬、责任过硬、能力过硬和作风过硬，全面建设人民满意的政府。一是自觉把人民二字刻在心上。人民是我们的衣食父母，为民服务是我们的天职。我们将始终保持对人民的赤子之心，自觉以百姓之心为心，以造福人民为最大政绩，做到利民之事、丝发必兴。二是自觉把实干担当抓在手上。实干推动发展，实干赢得未来，幸福都是奋斗出来的。我们要把践行“三严三实”贯穿于全部工作生活中，养成一种习惯、化为一种境界，持续改作风、提效能，注重调查研究、真抓实干；加大督查督办力度，从严整肃庸政懒政怠政行为，驰而不息反“四风”，为改革者鼓劲、为实干者撑腰、为担当者担当、为负责者负责，确保各项任务落到实处、见到实效。三是自觉把法治之责扛在肩上。加强法治政府建设，自觉接受人大及其常委会的法律监督和政协的民主监督，主动接受司法监督和舆论监督，认真办理人大代表建议和政协提案，广泛听取民主党派、工商联、无党派人士和各人民团体的意见。加强智库建设，推行“政府顾问”制度，建立行业产业决策咨询机制，不断提高政府工作法治化、专业化水平。四是自觉把廉洁要求顶在头上。为政，廉为首；为人，德为先。我们要向廉洁自律的高标准看齐，做到心有所戒、行有所止，守住底线、不碰高压线，严肃查处腐败行为，不断维护和巩固风清气正的政治生态。

　　各位代表！民生期许殷殷，奋斗未有穷期。时代是出卷人，我们是答卷人，人民是阅卷人，让我们更加紧密地团结在以习近平同志为核心的党中央周围，在市委的坚强领导下，大力弘扬跨越时空的井冈山精神，解放思想、改革创新，担当实干、马上就办，为实现“三个走在前列”“两大战略任务”、谱写新时代中国特色社会主义吉安篇章而不懈奋斗！
